# More than carbon sequestration: Biophysical climate benefits of restored savanna woodlands

**DOI:** 10.1038/srep29194

**Published:** 2016-07-04

**Authors:** Jozef I. Syktus, Clive A. McAlpine

**Affiliations:** 1 The University of Queensland, Global Change Institute, Brisbane 4072, Australia; 2 The University of Queensland, School of Geography, Planning and Environmental Management, Brisbane 4072, Australia

## Abstract

Deforestation and climate change are interconnected and represent major environmental challenges. Here, we explore the capacity of regional-scale restoration of marginal agricultural lands to savanna woodlands in Australia to reduce warming and drying resulting from increased concentration of greenhouse gases. We show that restoration triggers a positive feedback loop between the land surface and the atmosphere, characterised by increased evaporative fraction, eddy dissipation and turbulent mixing in the boundary-layer resulting in enhanced cloud formation and precipitation over the restored regions. The increased evapotranspiration results from the capacity deep-rooted woody vegetation to access soil moisture. As a consequence, the increase in precipitation provides additional moisture to soil and trees, thus reinforcing the positive feedback loop. Restoration reduced the rate of warming and drying under the transient increase in the radiative forcing of greenhouse gas emissions (RCP8.5). At the continental scale, average summer warming for all land areas was reduced by 0.18 ^o^C from 4.1 ^o^C for the period 2056–2075 compared to 1986–2005. For the restored regions (representing 20% of Australia), the averaged surface temperature increase was 3.2 °C which is 0.82 °C cooler compared to agricultural landscapes. Further, there was reduction of 12% in the summer drying of the near-surface soil for the restored regions.

Current rates of deforestation^1^,^2^,^3^ and global warming^4^, if unabated, will severely degrade the biosphere^5^. Forests are well recognised for their potential to mitigate climate change through carbon sequestration^6^. Less attention is given to their biophysical role in the energy and water cycles, and their capacity to regulate the regional climate^7^,^8^,^9^,^10^,^11^. At a regional scale, forest cover influences land-surface properties, including evapotranspiration, albedo and surface roughness, which affect the magnitude and form of energy transfer to the atmosphere^11^,^12^,^13^,^14^,^15^. By altering the fluxes of heat, momentum and moisture exchanges between the land surface and the lower atmosphere, forests affect climate.

Empirical evidence shows that tropical and boreal forests have divergent biophysical effects due to their dominant evapotranspiration and albedo effects respectively^9^,^10^. In general, forests evaporate more water than any other vegetation type – up to 10 times more than herbaceous vegetation^8^. Alkama and Cescatti^10^, using Earth Observations of global forest cover and land surface temperatures, showed evapotranspiration is the key biophysical process impacted by deforestation. The strongest sensitivity to loss of forest and other woody vegetation cover is in arid and semi-arid regions, followed by temperate, tropical, and boreal biomes^10^. In the lower latitudes, forests play an important role in regulating climate at the regional scale by enhancing the partitioning of energy into latent heat, thereby impacting moisture recycling, convective precipitation and cloud formation^9^,^10^,^11^,^12^,^13^,^14^,^15^,^16^,^17^,^18^. This ability to maintain evaporative cooling of the land surface is a key biophysical process by which forests regulate the regional climate 9,10,11,12,13,14. Jackson *et al*.^13^ argue that avoided deforestation, forest restoration and afforestation in tropical regions, provides the greatest climate benefits because carbon storage^19^,^20^ and biophysics align to cool the climate.

A number of studies using climate models have investigated the potential climate impacts of reforestation and afforestation^21^,^22^,^23^,^24^,^25^,^26^. Devaraju *et al*.^21^ show the biophysical and biochemical effects of land-use changes on climate differ in their spatial (latitudinal) influence. Biophysical effects are stronger at regional scales, whereas biochemical effects have global impacts. Latitude-specific land-use change modelling by Bala *et al*.^22^ show that afforestation in the tropics would be beneficial in mitigating global-scale warming but would have an opposite impact if implemented at high latitudes. A global study by Arora and Montenegro^23^ of the climate impact of complete and partial (50%) afforestation of croplands resulted in a reduction of warming of 0.45 °C and 0.25 °C respectively for the period 2081–2100 under the SRES A2 emissions scenario. Warming reductions per unit of afforested area were three times higher in tropical and sub-tropical regions than in boreal and northern temperate regions^23^. Galos^26^ used a regional climate model under the SRES A2 scenario to show that afforestation reduced warming of summer temperatures by 15–20% and halved the rainfall reduction due to global warming in the mid-latitude of Central Europe. The capacity of afforestation to impact regional climate is further demonstrated by the 62 million hectares of afforestation in China (15–55°N), which reduce daytime land-surface temperatures by 1.1 ± 0.5 °C due to increased evapotranspiration^27^. However, the net impact on temperature is influenced by the net difference between nighttime warming and daytime cooling, with nighttime warming increasing with latitude and decreasing with rainfall.

Here we evaluate the potential climate benefits of the regional-scale restoration of savanna woodlands in Australia under a global warming scenario. We use a high-resolution climate model to better resolve the influence of the spatial heterogeneity of soils and vegetation on regional climate processes. We focus on Australia, as a representative semi-arid region with highly-transformed landscapes^28^ and large areas of economically marginal agricultural land^29^. Historically, over 15% of the Australian continent has been converted to intensive cropping and livestock pastures. Many of the converted areas occur on the less fertile soils and are impacted by highly variable rainfall^29^,^30^. As a consequence, the economic viability of many agricultural landscapes is marginal, and is likely to decrease further as the climate becomes hotter and drier^31^,^32^,^33^,^34^. Since 2000, the rate of land clearing has slowed through the implementation of vegetation management legislation; however, these regulations are being relaxed in recent years, as the Australian and state governments seek to expand agricultural production to capitalise on the global demand for food commodities. There is increased pressure to expand the agro-pastoral frontier into intact regions of Northern Australia. A recent economic analysis of future land use options for Australia shows that large areas of economically marginal agricultural lands would have better economic and environmental return if used for carbon sequestration and other related ecosystem services^35^,^36^.

## Results

**Figure 1:**
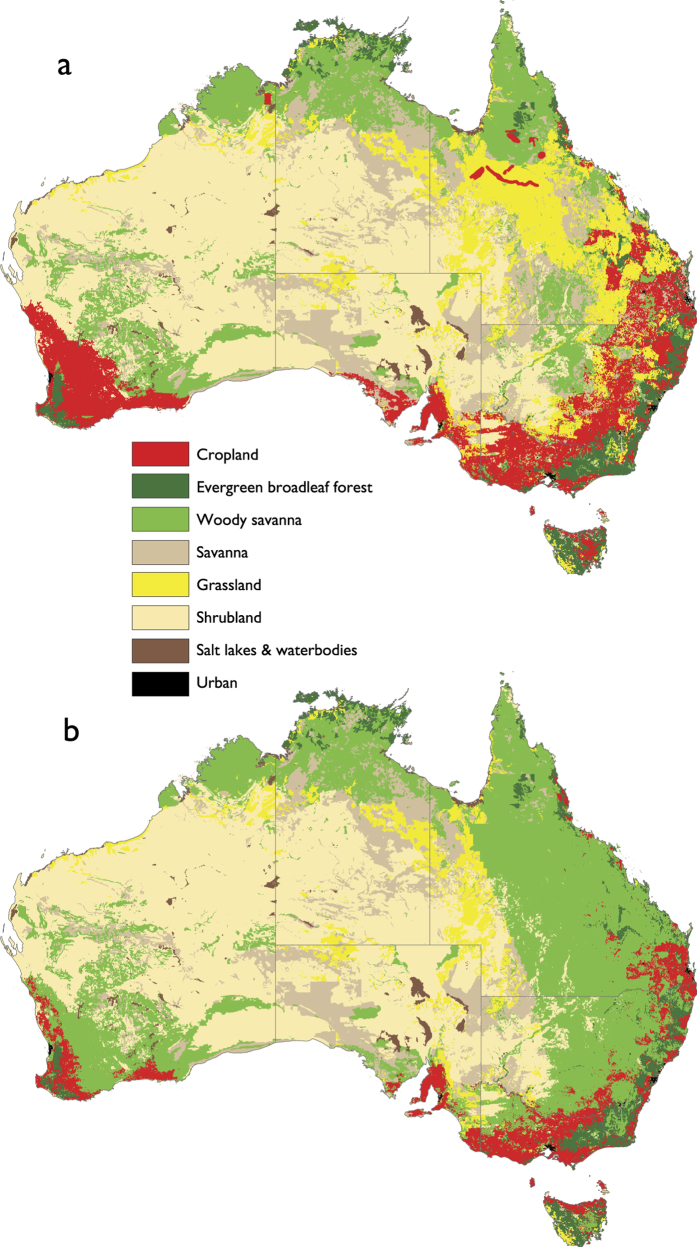
Land-use scenarios representative of: (**a**) Maximum Crops - maximum extent of crops and pastures; (**b**) Partial Restoration - maintaining crops and pastures on highly productive lands only. This figure was created using ArcGIS Version 10.3 (ESRI Inc., Redlands, CA, http://www.esri.com/software/arcgis).

We evaluated the climate impacts of two contrasting land-use scenarios for Australia (Fig. 1). The scenarios were representative of: i) the expansion of dryland crops and pastures (Maximum Crops, Fig. 1a), and ii) the maintenance of crops and pastures only on economically productive lands and the restoration of economically marginal lands^31^ to woodlands (Partial Restoration, Fig. 1b). The Maximum Crop scenario represents maintaining cropping and livestock pastures on historically-converted landscapes in southwest and eastern Australia, and an area of planned expansion in northern Australia. The Partial Restoration scenario represents maintaining high-production agricultural land (annual profit > $100/ha)^29^ and restoring the remaining less-profitable cropping and livestock pastures to woodlands.

The development of land-use scenarios is supported by recent assessments of the future land use options (up to 2050) for Australia based on the evaluation of sustainability of economic returns and provision of ecosystem services^35^,^36^,^37^. The analysis found substantial potential for land use transition from agriculture to carbon plantings, environmental plantings and biofuels cropping. With stronger abatement incentives, restoration of woody vegetation out-competes other land-uses over large parts of current grazing and dryland cropping lands in eastern Australia and southwest Western Australia^35^. The economic incentive for transition to carbon plantings was highest over the beef grazing areas of Queensland and northern New South Wales^35^ where woody vegetation has the capacity to passively regenerate in the absence of repeat clearing disturbance^38^,^39^.

The key difference between the land-use scenarios was the areal extent of agricultural lands versus woody savannas (Table 1). Under the Maximum Crops scenario, 24% of the Australian continent (excluding Tasmania) is used for cropping and livestock pastures, while woody savannas represent 20%. For the Partial Restoration Scenario, the area of crops and pastures decreased to 11% and woody savanna increased to 35%. The increase in the area of woody savannas under the Partial Restoration scenario was highest in Queensland and southeast Australia combined (Table 1). Although the increase in the area of woody savannas for Western Australia was relatively low, this was concentrated in southwestern corner of the state and represents a large proportional change (Fig. 1). At the continental scale, the restoration resulted in a 15% increase in the area of woody savannas. Some savanna and grassland ecosystems in western Queensland and New South Wales were also reclassified to woody savanna under the Partial Restoration scenario (Fig. 1b). In total 20% of continental Australia was covered by savanna woodland.

The key difference in the land-surface characteristics between the scenarios used in the model experiments was the land-use distribution and leaf area index (LAI). To quantify the biophysical impact of these contrasting scenarios on regional climate, we applied the CSIRO Conformal Cubic Atmospheric Model (CCAM, see Model Description in the Methods section)^40^,^41^, a variable resolution global climate model, with an enhanced spatial resolution of 20 km over the Australian region. An ensemble of three simulations for each land-use scenario for the period 2021–2076 was completed. The time-varying changes in greenhouse gases, aerosols and ozone concentration following the RCP8.5 emissions^42^ were used in all simulations. The land use scenarios were invariant in time and represented average climatological state of two contrasting land-surface characteristics. As a result, the only difference between the two sets of simulations was the land-use scenarios (see Methods section).

### The impact of restoration on surface climate

**Figure 2:**
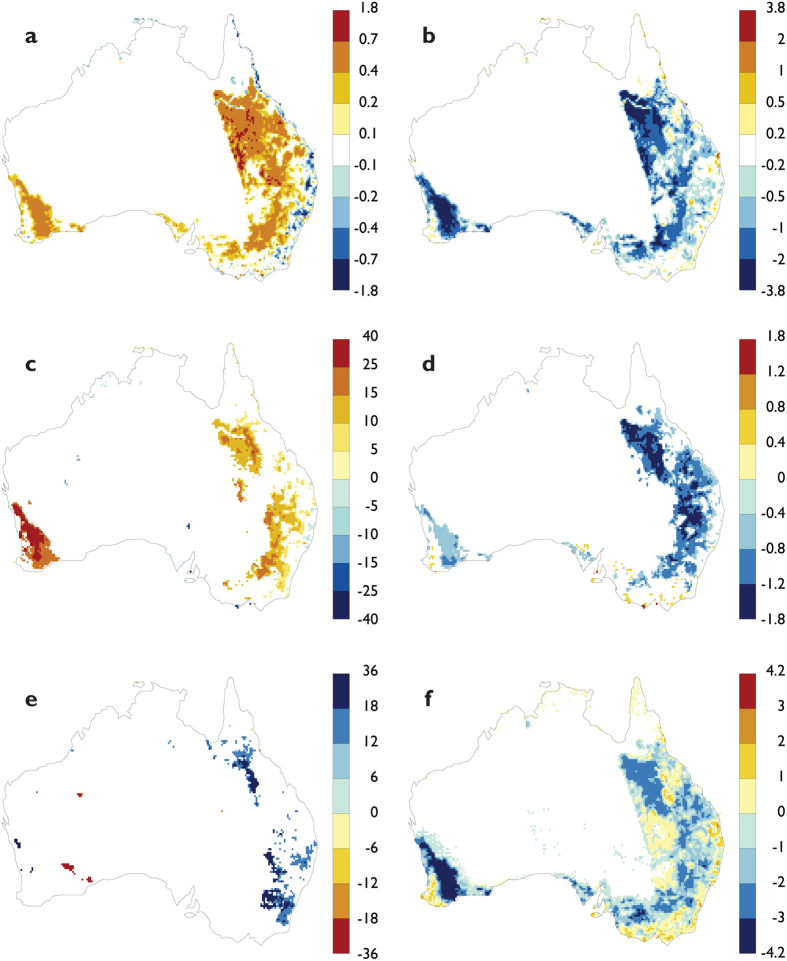
Change in land surface characteristics and surface climate between the Partial Restoration and Maximum Crops land use scenarios for the summer season (DJF). Differences in the ensemble average for the period 2023–2076 for: (**a**) leaf area index (LAI) (dimensionless); (**b**) surface albedo (×100); (**c**) latent heat-flux %; (**d**) surface temperature, °C; (**e**) rainfall (note the reverse colour scale), %; (**f**) surface wind speed at 10 metres, m/s. Results are shown for P < 0.05 significance level using bootstrap Monte-Carlo resampling. This figure was created using Ferret Version 6.93 (NOAA/PMEL, http://www.ferret.noaa.gov/Ferret/).

The restoration of marginal agricultural lands with savanna woodlands had a substantial (see Analysis in the Methods section) impact on the regional climate of Australia. The analysis shows the impacts of restoration are most pronounced during summer season (DJF) and weakest during the winter season (JJA). The changes for the summer conditions are derived from the ensemble-average of three simulations for each land-use scenario for the 2023–2076 periods (Fig. 2). The annually-averaged results show a similar magnitude and pattern of change to the summer season (see Fig. 1S). Here we present only statistically significant differences at P < 0.05 for summer. In general, the statistically significant changes in climate variables were largely confined to the areas of restored vegetation.

The key difference in land surface characteristics between the Partial Restoration and Maximum Crop scenarios was a higher leaf-area index (LAI) (Fig. 2a), decreased surface albedo (Fig. 2b) and increased surface roughness. These prescribed changes in surface characteristics were the main drivers of the climate response for each scenario. The increase in tree cover and the associated higher LAI resulted in an enhanced latent heat flux from the land surface to the atmosphere by 5–40%, with the strongest proportional increase in southwest Australia (Fig. 2c). The area-averaged latent heat over all restored regions (Fig. 2a), that cover one fifth of continental Australia, increased by 10%. For the whole continent, the increase was 2.6%. The analysis of the surface energy balance showed an increased partitioning of latent heat relative to sensible heat. The increase in latent heating dominated the climate response and cancelled the impact of increased short-wave radiation due to the decrease in albedo (Fig. 2b). On average, the albedo decreased by 1% over all restored regions. The resulting stronger evaporative cooling led to lower surface temperatures, with a cooling of 0.66 °C averaged over all restored areas (Fig. 2d). Regionally, the cooling was most pronounced over the restored areas of Queensland (0.76 °C) and least pronounced over the restored areas of southwest Australia (0.49 °C). This response is consistent with other studies showing that evaporative cooling is the dominant biophysical process associated with forest cover change^9^,^10^,^44^,^45^,^46^. The increased moisture availability associated with an enhanced latent heat flux from the restored woodlands led to an increase in rainfall by 9.9% averaged over all restored areas (Fig. 2e). At the continental scale, area-average rainfall increase over all land areas was ~2.6%. Regionally, the rainfall increase aggregated over the restored areas was strongest in southwest Australia (15.2%) and southeast Australia (11.8%), and less in Queensland (8.7%). The increased surface roughness associated with the restored woody vegetation resulted in a wind speed reduction by 1.4 m/s averaged over all restored areas (Fig. 2f). This was most pronounced over southwest Australia (2.6 m/s) and least over Queensland (1.1 m/s).

### The impact of restoration on cloud formation and rainfall

**Figure 3:**
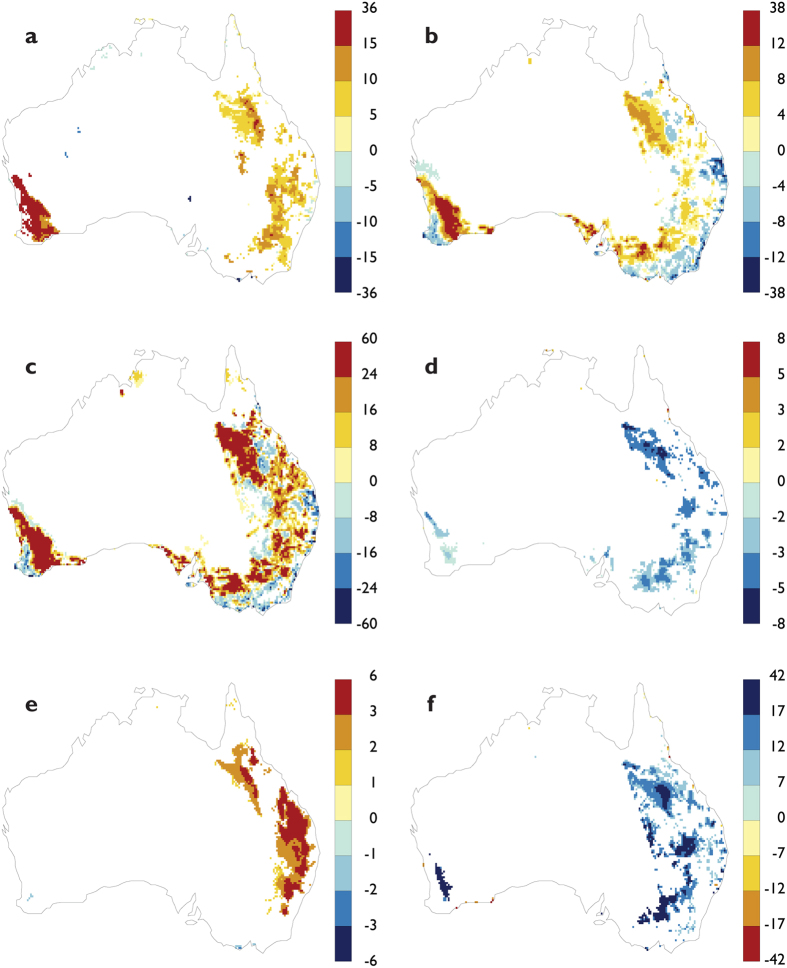
Changes in boundary layer and cloud formation processes between the Partial Restoration and Maximum Crops land use scenarios for the summer (DJF) climate. Differences in the ensemble average for the period 2023–2076 for: (**a**) evaporative fraction (proportion of latent heat to sensible heat), %; (**b**) turbulence kinetic energy averaged over the first 900 m of the lower-atmosphere, %; (**c**) eddy dissipation rate averaged over the first 900 m of the lower-atmosphere, %; (**d**) cloud base height, hPa; (**e**) low cloud cover, %; (**f**) convective rainfall (note the reverse colour scale), %. Results are shown for P < 0.05 significance level using bootstrap Monte-Carlo resampling. This figure was created using Ferret Version 6.93 (NOAA/PMEL, http://www.ferret.noaa.gov/Ferret/).

The restoration of savanna woodlands significantly impacted on the boundary layer, cloud formation and precipitation processes (Fig. 3). The increased access to soil moisture by deep-rooted woodlands altered the partitioning of surface energy, with an increase in evaporative fraction (proportion of latent heat flux relative to sensible heat flux) of 7.1% averaged over all restored regions (Fig. 3a). Averaged over the continental Australia, the increase in evaporative fraction was 2.2%. Turbulence is one of the most important physical processes in the boundary layer driving the vertical transport of water vapor, momentum and mass, which impacts cloud formation and precipitation processes in the lower atmosphere^47^. The prognostic calculation shows an increase in turbulence kinetic energy (TKE) in the lower atmosphere, with an average increase over all restored regions of 3.5% (Fig. 3b). The largest proportional increase, averaged over the restored regions, was 9.2% for southwest Western Australia. This increase in TKE resulted in an enhanced eddy dissipation rate in the atmospheric boundary layer (Fig. 3c). The average increase over all restored regions was 16% (Fig. 3b). The largest proportional increase over restored regions was 31% for southwest Western Australia and 14% for southeast Australia. The increase in turbulent motion impacted the entrainment and mixing of clouds, resulting a lowering of the cloud base by 2–8 hPa, predominantly over eastern Australia (Fig. 3d). On average, the cloud base decreased by 2 hPa over the restored areas. The enhanced mixing and associated changes in the boundary layer resulted in an increase in low cloud cover (Fig. 3e). This increase was concentrated over restored areas of eastern Australia with an average increase of 1.8%.

The enhanced cloud formation and associated boundary layer changes led to an increase in convective rainfall (Fig. 3f). On average, the convective rainfall increased by 11% over the restored areas and 3.5% over all land areas of continental Australia. The convective rainfall increase was more extensive and larger than the changes in total rainfall (Fig. 2e), which indicates that enhanced mixing and convective processes dominated the rainfall increase. The annually-averaged impacts of restoration on the boundary layer processes, cloud formation and precipitation showed a similar response (Supplementary Fig. 2).

Next, we assess the capacity of restoration to impact the rate of warming under the high emission RCP8.5 during the second half of the 21^st^ century.

### The capacity of restoration to reduce the regional impacts of global warming

**Figure 4:**
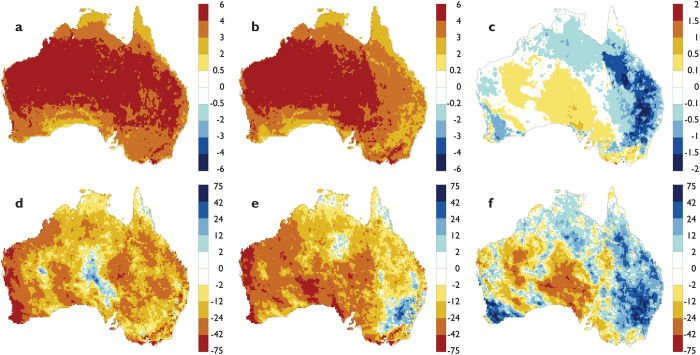
Changes in summer (DJF) surface temperature and near surface soil moisture for the 2056–2075 period relative to the 1986–2005 period. Change in surface temperature, °C for: (**a**) RCP8.5 with Maximum Crops; (**b**) RCP8.5 with Partial Restoration; (**c**) impact of restoration (**b** minus **a**). Change in near surface soil moisture, %; (**d**) RCP8.5 with Maximum Crops; (**e**) RCP8.5 for Partial Restoration; f, impact of restoration (**e** minus **d**). This figure was created using Ferret Version 6.93 (NOAA/PMEL, http://www.ferret.noaa.gov/Ferret/).

**Figure 5:**
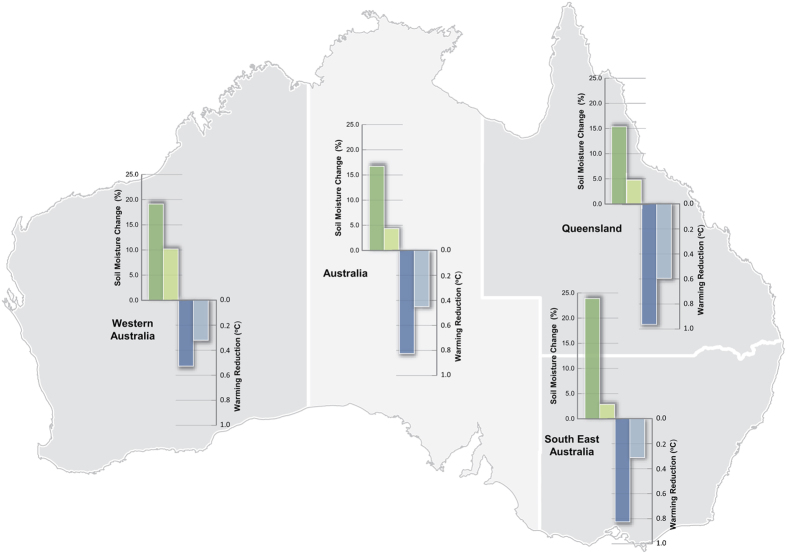
Summary of simulated changes in surface temperatures and near surface soil moisture between Partial Restoration and Maximum Crop for the 2056–2075 period under RCP8.5. Area average changes over restored regions of Australia (all restored regions), Western Australia, Queensland and southeast Australia are presented for summer (darker colour) and annual (lighter colour). Figure was created using Adobe Illustrator Version CC2015(19.2.0), (http://www.adobe.com/au/products/illustrator.html#).

The Partial Restoration scenario resulted in substantially different regional climate changes during the 2056–2075 period. Under the Maximum Crop scenario, the mean summer surface temperature averaged over all restored regions (Fig. 2a) increased by ~4.0 °C for the 2056–2075 period compared to the 1986–2005 period (Fig. 4a). Under the Partial Restoration scenario (Fig. 4b), the average summer temperature increased was 3.2 °C, which represents a 21% reduction in the rate of warming under RCP8.5 as a result of restoration (Fig. 4c). Over all land areas of the continent, area-average warming was reduced by ~5% (0.18 °C). At the regional scale, the reduction in the area-averaged rate of warming was strongest over the restored areas of eastern Australia (0.91 °C) while the reduction was 0.53 °C for southwest Western Australia (Fig. 5).

In addition to a reduced rate of warming, restoration had a positive impact on surface soil moisture. Under the Maximum Crop scenario, the average summer soil moisture averaged over all restored regions decreased by 23.4% for the 2056–2075 period compared to the 1986–2005 period (Fig. 4d). In contrast, the area-averaged summer soil moisture decreased by 11.1% for the Partial Restoration scenario (Fig. 4e). The net benefit of restoration on soil moisture (Fig. 4f) averaged over all restored areas was ~16.7% (Fig. 5). This benefit was strongest over the restored regions of southeast Australia (24%) and southwest Western Australia (19%). These results demonstrate that regional-scale restoration of economically marginal agricultural lands to savanna woodlands has the capacity to reduce the magnitude of regional warming and drying under future high-emissions of greenhouse gases as represented by RCP8.5.

## Discussion

In this study, we used a high resolution coupled atmosphere-land surface model to evaluate the capacity of the restoration of Australia’s economically-marginal agricultural lands to reduce the impact of global warming due to the increased concentration of greenhouse gases. We applied an ensemble approach and CMIP5 radiative forcing which included changes in greenhouse gases, ozone and aerosols^42^,^43^. Using land-use scenarios and the high-emission RCP8.5 scenario, we demonstrate that regional-scale restoration has the capacity to moderate the warming and drying at the regional scale.

The results demonstrate that the restoration of savanna woodlands maintains a positive feedback loop between the land surface and the atmosphere, characterised by increased evaporative fraction and increased turbulent mixing in the boundary layer. These processes resulted in enhanced cloud formation and convective precipitation over the restored areas. The maintenance of a positive feedback loop between the land surface and the atmosphere is a direct consequence of the ability of deep-rooted trees to extract moisture from the whole soil profile and maintain evapotranspiration during hot and dry conditions^17^,^44^. In contrast, shallow-rooted rainfed crops and pastures have a limited capacity to maintain this positive feedback. A key difference between the representation of woody savannas and crops/pastures in the CCAM-CABLE soil profile is the root biomass, which is tenfold larger for woody savannas. In addition, crops have 70% of roots in the top 30 cm of soil profile whereas woody savannas have 43% of roots in deeper (>0.3–4.5 m) parts of the soil profile^48^.

This biophysical function of restored vegetation exerts a moderating influence on the regional climate, especially during the warm season, resulting in a cooler and a moister climate. In response to restoration, evaporative fraction increased which was more important than albedo decrease, highlighting the ecological function of trees in maintaining moisture supply to the atmosphere and thereby enhancing cloud formation and convective rainfall^8^,^11^,^12^,^18^,^40^. In turn, the increased rainfall provides additional moisture to the soils and vegetation, reinforcing the positive biophysical feedbacks. Our modelling results are consistent with observation-based analysis of historical conversion of native vegetation to crops and pastures in southwest Australia^49^. This study compared the impact of adjoining contrasting land surfaces along the “Bunny Fence” on cloud formation and energy fluxes, showing increased latent heat fluxes and higher available soil moisture over native vegetation compared to agricultural areas during summer. The increased moisture availability enhanced cumulus cloudiness over native vegetation.

The results of our simulations show that these biophysical feedbacks resulting from restoration have the potential to exert a moderating influence on the rate of warming and drying under the high emission RCP8.5. However, they are potentially model-dependent and additional modelling studies would help to check the robustness of our findings. The results are consistent with recent global observational^9^,^10^,^17^ evidence which shows that semi-arid vegetation exerts a significant influence on climate which is driven by evapotranspiration^10^,^50^.

As humanity continues to struggle with finding a sustainable balance between the economy and the environment, there is a need for a stronger focus on integrating land-use and climate-change policies. Current land management in tropical and semi-arid regions is largely focused on a “business-as-usual” approach of achieving short to medium-term economic returns^5^,^51^,^52^ associated with expansion of agricultural production and resource extraction^1^,^2^. This leads to the loss of important regional-scale biophysical climate services^13^,^14^, as well as likely negative impacts on carbon sequestration, biodiversity and key ecosystem services^53^,^54^. These management practices tend to reinforce regional warming and drying^31^,^32^,^33^,^55^,^56^,^57^. Our results support an alternative, more sustainable pathway, which seeks to intensify agriculture on the highly productive lands to meet the demand for food production while restoring savanna woodlands on marginal agricultural lands^35^,^36^,^37^,^52^,^53^. The results highlight the potential of savanna woodland restoration for regional climate protection^6^,^13^,^14^,^16^, when combined with the reduction in global greenhouse gas emissions. The economic rationale for restoration is likely to become stronger as the price on carbon increases^35^,^36^,^37^. Large-scale restoration is ecologically and economically feasible^35^,^36^,^37^ as large areas of Australia’s savanna woodlands have the capacity to passively regenerate^38^,^39^,^58^. As the climate becomes hotter and drier, and land-use pressures increase, the imperative for conserving and restoring woody vegetation will become more urgent^59^. We conclude that the restoration of Australia’s marginal agricultural lands to woody savannas has a capacity to substantially reduce the magnitude of warming and drying over restored regions under high-emission scenario during the 21^st^ century.

## Methods

### Land-use scenarios

Two land-use scenarios were developed for Australia based on national vegetation cover and land-use mapping^60^,^61^. This high-resolution national dataset was reclassified to represent distinct land-use classes using the IGBP land use/land cover classification as used in the CSIRO CCAM-CABLE climate model. The first scenario represented the expansion of dryland crops and pastures (Maximum Crops, Fig. 1a). The second scenario represented the retention of crops and pastures on economically productive lands and the restoration of economically marginal lands to savanna woodlands (Partial Restoration, Fig. 1b). The reduction of the area of crops and scenarios was based on agricultural profitability mapping^29^, where annual profit less than $100/ha was used to define economically-marginal agricultural lands. The Partial Restoration scenario represents maintaining high-production agricultural land (annual profit > $100/ha)^29^ and restoring the remaining less-profitable cropping and livestock pastures to woodlands.

The first scenario focused on the extensive use of Australia’s landscapes for crops and livestock pastures (Fig. 1a). It was designed to represent the current distribution of crops and pastures and plausible future expansion. The second scenario represents maintaining high-production agricultural land (annual profit > $100/ha)^29^ and restoring the remaining less-profitable crop/pastureland to woodlands (Fig. 1b). The vegetation cover of the extensive arid regions and conservation areas was left unchanged for both scenarios. Global high resolution Leaf Area Index (LAI)^62^, averaged for the 2000–2009 period, based on MODIS C5 was used to derive monthly LAI corresponding to the land use/land cover classification for each scenario. Each scenario was represented by distinct IGBP land use/land cover classes (Fig. 1) and monthly LAI data (Fig. 2a). The two land use scenarios were invariant in time and as such represent the average climatological state of two contrasting land-surface characteristics.

### Model description

We used the stretched version of global variable resolution Conformal-Cubic Atmospheric Model (CCAM)^40^,^41^, coupled to the CSIRO Atmosphere Biosphere Land Exchange (CABLE 1.8)^63^,^64^. This variable-resolution global model with advanced dynamical core is numerically efficient and provides an attractive alternative to limited-area regional models for dynamical downscaling. Unlike regional models, CCAM is dynamically consistent globally and avoids problems with specifying lateral-boundary forcing^65^. CCAM is formulated on a quasi-uniform grid derived by projecting the panels of a cube onto the surface of the Earth. In this study, the model was run in stretched mode with 20 km horizontal resolution over Australia and with 27 atmospheric vertical levels and six soil levels. CCAM contains a comprehensive set of physical parameterizations as described by Thevakaran^65^. In CCAM, the land surface processes are represented by the CABLE biosphere land exchange model. CABLE simulates the exchange of CO_2_, radiation, heat, water and momentum fluxes between the land surface and atmosphere, and is composed of five main sub-models: (1) the radiation sub-model estimates the radiation transfer and absorption by both sunlit and shaded leaves and by soil surface in the visible, near infrared and thermal radiation, and also the surface albedo for visible and near infrared radiation^66^; (2) the surface flux sub-model estimates the coupled transpiration, stomatal conductance, photosynthesis and partitioning of net available energy between latent and sensible heat of sunlit and shaded leaves^66^. Photosynthesis is calculated for both C3 and C4 plants; (3) the canopy micrometeorology sub-model describes the surface roughness length, zero plane displacement height, and aerodynamic resistance from the reference height to the air within the canopy or to the soil surface^67^; (4) the soil and snow sub-models compute the heat and water fluxes within each of the six soil layers and three snowpack layers, snow age, snow density and snow depth, and snow covered surface albedo. Soil moisture is calculated according to the Richards’ equation and the heat conduction equation is used to obtain soil temperature^63^,^64^; and (5) the ecosystem carbon module, which estimates respiration of stem, root and soil organic carbon decomposition^68^.

### Experimental design

The CCAM model was used to evaluate the impact of the two contrasting climatologically-averaged land-use scenarios (Fig. 1) under transient changes in radiative forcing^42^,^43^ such as greenhouse-gas and aerosols following RCP8.5 emissions. An ensemble of three simulations for each scenario was completed for the period 2021–2076 at a 20 km spatial resolution over the Australian region. The simulations started with different initial conditions, and used bias-corrected SSTs and sea-ice cover taken from the equivalent RCP8.5 simulations with the CSIRO Mk3.6 climate model^43^. To contrast differences in the warming between 2056–2075 and 1986–2005 under the two contrasting land-use scenarios (Fig. 4), we used data from historical simulations with CCAM for the 1960–2005 period. The historical simulations used recent land use data, historical radiative forcing and bias-corrected sea-surface temperatures taken from the CMIP5 simulations with the CSIRO Mk3.6 global climate model^43^. The high-spatial resolution CCAM-CABLE model was used to improve the representation of topographic features, soil and landscape heterogeneity and regional land surface processes.

### Analysis

We used monthly output from CCAM simulations to produce multi-year climatological averages for annual and seasonal conditions for the period 2023–2076. The first two years of simulations were discarded to allow for model spin-up. Using these data, we analysed differences in the climatological response between the two simulated scenarios. In presenting the results, we focused on providing area-averaged values over all restored regions of Australia, which covered 20% of continental Australia (Fig. 2a). Regionally, we show area-averaged values over the restored regions of Queensland, southeast Australia (New South Wales-Victoria combined) and Western Australia. Results are also shown as area-averaged values for all land areas of continental Australia (excluding Tasmania). The statistical significance of the simulated differences was assessed using non-parametric bootstrap resampling^69^. This approach is considered to be a robust test for assessing the statistical significance of the climate modelling results. For each set of simulations, we calculated the ensemble average seasonal mean of various climate variables such as temperature, precipitation and wind speed and analysed the statistical difference using bootstrapping at 95% confidence level (P < 0.05). We compared the sensitivity of statistical tests commonly used in climate modelling experiments such as the student T-test and the Kolmogorov-Smirnov (KS-test) to the bootstrapping approach. All tests showed similar results. In our study, N = 162 (3 ensembles × 54 years of model integration), with N = 500 bootstrap samples conducted to test for statistical significance.

## Additional Information

**How to cite this article**: Syktus, J. I. and McAlpine, C. A. More than carbon sequestration: Biophysical climate benefits of restored savanna woodlands. *Sci. Rep.* **6**, 29194; doi: 10.1038/srep29194 (2016).

## Supplementary Material

Supplementary Information

## Figures and Tables

**Table 1:** Summary statistics of the area of woody savannas.

Region	Maximum Crop Scenario	Partial Restoration Scenario	Net increase in area of woody savanna	% of total land area impacted by restoration
Australia	1,514,755	2,674,238	1,159,483	15.1
Queensland	428,275	1,153,993	725,718	42.1
Southeast Australia	139,612	476,562	336,950	32.8
Western Australia	482,781	651,283	168,502	6.7

For the Maximum Cropping and Partial Restoration scenarios (units km^2^) and the percent of the total land area restored to woody savannas.
