# Supplementary material for: More than carbon sequestration: Biophysical climate benefits of restored savanna woodlands
Source: Sci Rep. 2016 Jul 4;6:29194. doi: 10.1038/srep29194 (PMC4931580; doi:10.1038/srep29194)
Supplement: Supplementary Information [file srep29194-s1.doc]

**More than carbon sequestration: Biophysical climate benefits of restored savanna woodlands**

**Jozef Syktus^1^ and Clive McAlpine^2^**

^1^Global Change Institute, University of Queensland, Brisbane 4072, Australia

^2^School of Geography, Planning and Environmental Management, University of Queensland, Brisbane 4072, Australia

^2^ For correspondence. E-mail: [c.mcalpine@uq.edu.au](../../../../../c.mcalpine@uq.edu.au)


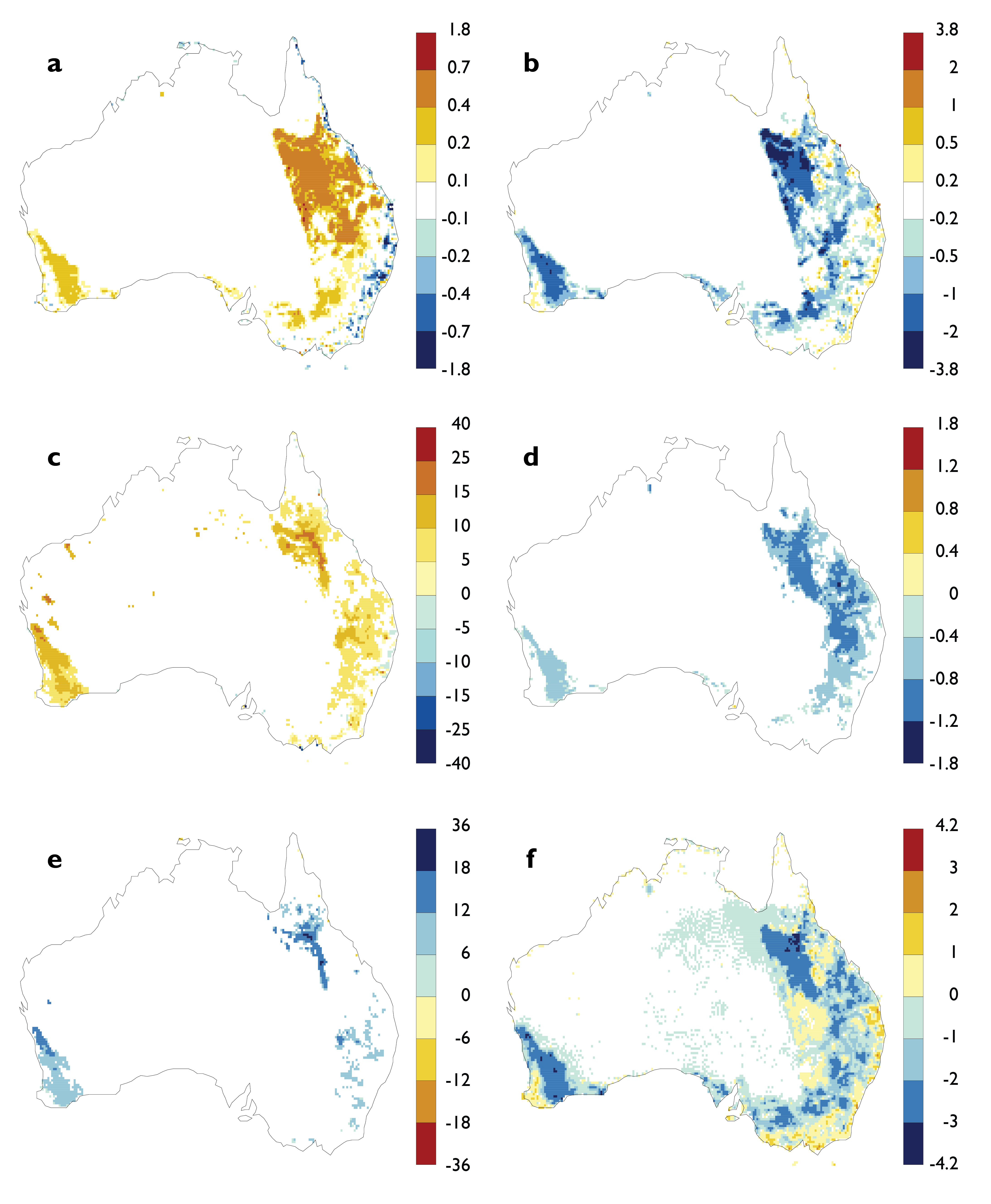


**Figure 1S| Annual changes in land surface characteristics and surface climate** between the Partial Restoration and Maximum Crops land use scenarios. Differences in the ensemble average for the period 2023–2076 for: a, leaf area index (LAI) (dimensionless); b, surface albedo (x 100); c, latent heat-flux, %; d, surface temperature, ^o^C; e, rainfall (note the reverse colour scale), %; f, surface wind speed at 10 metres, m/s. Results are shown for P<0.05 significance level using bootstrap Monte-Carlo resampling. This figure was created using Ferret Version 6.93 (NOAA/PMEL, <http://www.ferret.noaa.gov/Ferret/>).

**
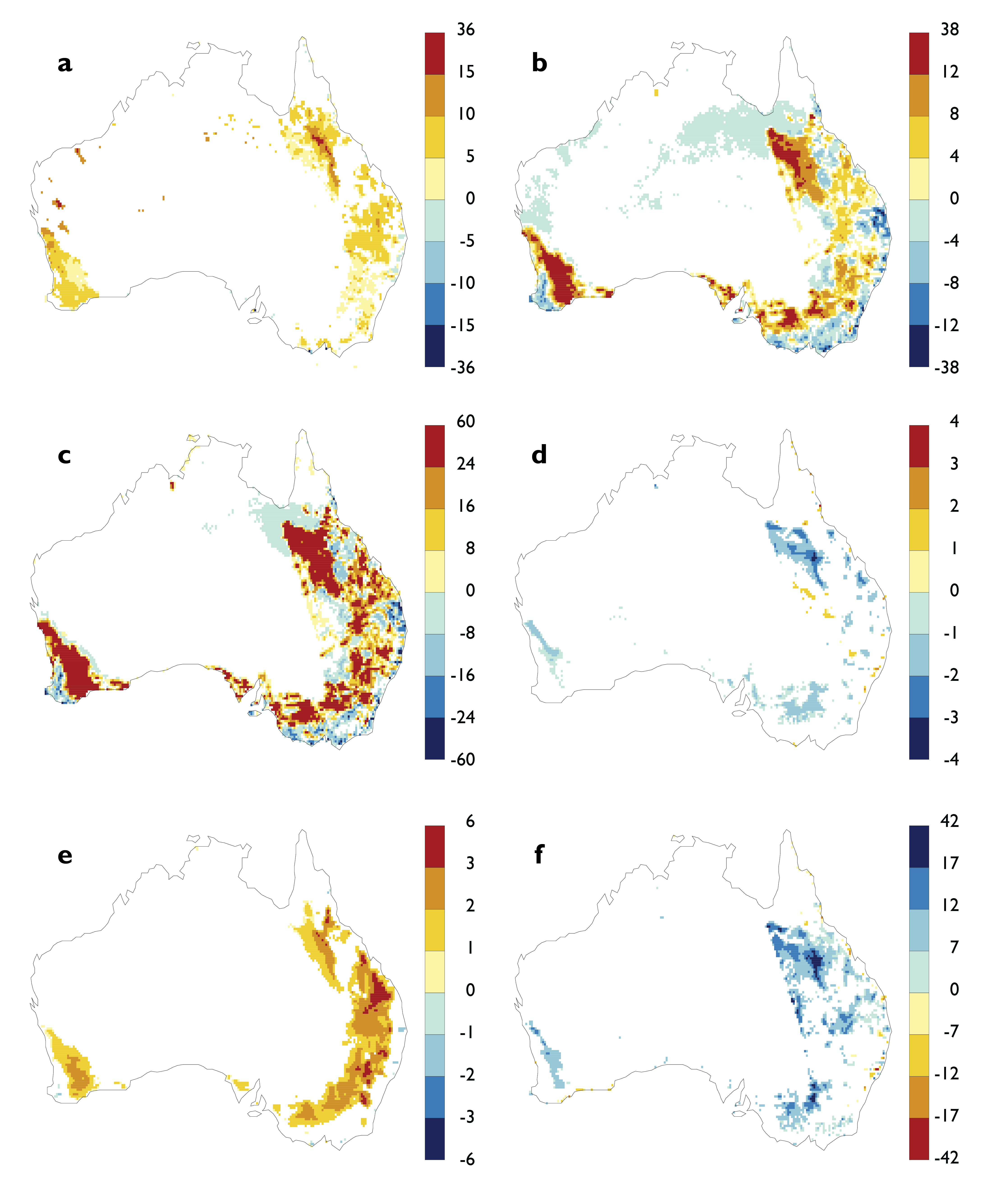
**

**Figure 2S| Annual changes in boundary layer and cloud formation processes** between the Partial Restoration and Maximum Crops land use scenarios. Differences in the ensemble average for the period 2023–2076 for: a, evaporative fraction (proportion of latent heat to sensible heat), %; b, turbulence kinetic energy averaged over the first 900 m of the lower-atmosphere, %; c, eddy dissipation rate averaged over the first 900 m of the lower-atmosphere, %; d, cloud base height, hPa; e, low cloud cover, %; f, convective rainfall (note the reverse colour scale), %. Results are shown for P<0.05 significance level using bootstrap Monte-Carlo resampling. This figure was created using Ferret Version 6.93 (NOAA/PMEL, <http://www.ferret.noaa.gov/Ferret/>).
